# WIPI2 enhances the vulnerability of colorectal cancer cells to erastin via bioinformatics analysis and experimental verification

**DOI:** 10.3389/fonc.2023.1146617

**Published:** 2023-05-03

**Authors:** Liying Yu, Yan Luo, Xile Ding, Miaomiao Tang, Huan Gao, Renfang Zhang, Mingfu Chen, Yuchen Liu, Qiongxia Chen, Yanli Ouyang, Xiang Wang, Hongyan Zhen

**Affiliations:** ^1^Department of Pathology and Pathophysiology, School of Medicine, Jianghan University, Wuhan, China; ^2^Cancer Institute, School of Medicine, Jianghan University, Wuhan, China; ^3^Wuhan Institute of Biomedical Sciences, School of Medicine, Jianghan University, Wuhan, China; ^4^Department of Pathology, Fifth Hospital in Wuhan, Wuhan, China; ^5^Department of Histology and Embryology, School of Medicine, Jianghan University, Wuhan, China

**Keywords:** WIPI2, colorectal cancer, ferroptosis, erastin, glutathione peroxidase 4

## Abstract

**Introduction:**

WD Repeat Domain Phosphoinositide Interacting 2 (WIPI2) is a WD repeat protein that interacts with phosphatidylinositol and regulates multiprotein complexes by providing a b-propeller platform for synchronous and reversible protein-protein interactions assembled proteins. Ferroptosis is a novel iron-dependent form of cell death. It is usually accompanied with the accumulation of membrane lipid peroxides. Our study is to focus on investigating the effect of WIPI2 on the growth and ferroptosis of colorectal cancer (CRC) cells and its potential mechanism.

**Methods:**

We analyzed the expression of WIPI2 in colorectal cancer versus normal tissues through The Cancer Genome Atlas (TCGA), and the relationship between clinical traits and WIPI2 expression and prognosis was assessed by univariate and multifactorial cox analysis. Next, we constructed the siRNAs targeting the WIPI2 sequence si-WIPI2 to further investigate the mechanism of WIPI2 in CRC cells through vitro experiments.

**Results:**

Public data from the TCGA platform showed that WIPI2 expression was significantly elevated in colorectal cancer tissues compared to paracancerous tissues, and high WIPI2 expressionpredicted poor prognosis for CRC patients. Moreover, we found that the knockdown of WIPI2 expression could inhibit the growth and proliferation of HCT116 and HT29 cells. Furthermore, we found that the expression level of ACSL4 decreased and that of GPX4 increased when WIPI2 was knocked down, suggesting that WIPI2 can potentially positively regulate CRC ferroptosis. Meanwhile, both NC and si groups were able to further inhibit cell growth activity, as well as increase WIPI2 and decrease GPX4 expression when treated with Erastin, but the rate of cell viability inhibition and the trend of protein changes were more significantly in the NC group than si groups, which indicated that Erastin induced CRC ferroptosis through the WIPI2/GPX4 pathway thereby enhancing the sensitivity of colorectal cancer cells to Erastin.

**Conclusions:**

Our study suggested that WIPI2 had a promotional effect on the growth of colorectal cancer cells, and it also played an important role in the ferroptosis pathway.

## Introduction

According to the latest statistical report from the International Agency for Research on Cancer (IARC) of the World Health Organization, there were about 9.96 million cancer deaths and 19.29 million new cancer diagnoses worldwide in 2020 (1). In recent years, with changes in people’s dietary habits, living environment and other conditions, among which the incidence and fatality rates have increased in colorectal cancer year by year, with its incidence rate ranking the third among all malignant tumors and mortality rate ranking the second (1). Today, the clinical therapy options for colorectal cancer primarily include surgery, radiotherapy, chemotherapy, immunotherapy and biologically targeted therapy (2). Nevertheless, due to the insidious onset of colorectal cancer and the lack of obvious early symptoms, many patients have progressed to the middle and late stages by the time they are diagnosed, and therefore the surgical efficacy is poor, and most patients have a low 5-year survival rate after surgery (3). Therefore, exploring the mechanisms of colorectal cancer development and finding more potential molecular markers involved in colorectal carcinogenesis are of great importance for early screening of colorectal cancer and the development of individualized treatment plans.

Compared to normal cells, tumor cells display higher iron requirements in order to adapt to overgrowth, and because of their need on iron, tumor cells are more vulnerable to iron-catalyzed death (4). In 2012, Stockwell proposed a new kind of programmed cell death (regulated cell death, RCD) mainly characterized by the accumulation of intracellular iron ions and lipid peroxides, which is different from both autophagy and apoptosis, and named it Ferroptosis (5).

Ferroptosis has now been extensively studied in various fields, where it is also closely associated with tumor development (6). Several studies have shown that it plays an important regulatory role in a variety of tumors such as liver, breast, pancreatic, colorectal, lung, ovarian and glioma, and can effectively inhibit tumor proliferation (7–9). Park et al. (10) demonstrated that bromelain can effectively inhibit the proliferation and metastasis of CRC cells by regulating ACSL4 levels, contributing to ferroptosis in K-ras mutant cell lines. Zhang et al. (11) reported that the benzopyranderivative 2-imino-6-methoxy-2H-chromene-3-carbothioamide (IMCA) could induce SLC7A11 downregulation as well as ROS accumulation through the AMPK/mTOR pathway, ultimately inducing ferroptosis in HCT116 and DLD-1 cells. In addition, unlike in other tumor cells, p53 can mediate the expression of SCL7A11 through a non-transcriptional mechanism in colorectal cancer cells, thereby affecting the anti-cancer activity of the ferroptosis inducer Erastin *in vivo *(12). In summary, these findings indicate that ferroptosis may regulate the development of colorectal cancer, but its specific mechanisms still need to be explored and studied in greater depth.

β-propellers that bind polyphosphoinositides (PROPPINs), known as WD repeat proteins that interact with phosphatidylinositol, are essential components of many important biological functions in mammalian cells. And they regulate the combinations of multiprotein complexes by providing a reversible protein-protein interaction β-propeller platform to regulate the assembly of multiprotein complexes. The WD repeat protein family consists of four major members, WIPI1, WIPI2, WDR45B/WIPI3 and WDR45/WIPI4/WDRX1, and their splice variants (13). In addition, human WIPIs genes are commonly expressed in normal somatic tissues, are highly expressed in skeletal and cardiac muscle, and almost all of the WIPI family of proteins have been found to be aberrantly expressed in different human tumors (13). Among them, WIPI2 (WD Repeat Domain, Phosphoinositide Interacting 2) is a protein-coding gene with a 7-bladed propeller structure containing a conserved motif that interacts with phospholipids (13). In recent years, it has been showed that WIPI2 may be strongly associated with tumor development. Such as, Gao et al. reported that CERS6-AS1 could promote pancreatic cancer cell proliferation and inhibit apoptosis through the miR-195-5p/WIPI2 axis (14). Liu et al. reported that BRG1 can regulate autophagy-dependent oxidative stress in the colon via WIPI2, thus exerting a regulatory role on inflammation-associated colon cancer (15). In addition, Liu et al. have also demonstrated that WIPI2 deficiency can inhibit the proliferation of HCC cells via the AMPK signaling pathway (16). However, most of the current studies on WIPI2 have focused on its regulatory role in autophagy, the interaction between WIPI2 and ferroptosis has not been reported yet.

Therefore, we sought to investigate whether WIPI2 may influence the development of colorectal cancer via the ferroptosis pathway and aimed to explore its associated molecular mechanisms.

## Materials and methods

### Extraction and analysis of the TCGA dataset

Sequencing data from colorectal cancer tissues were downloaded from The Cancer Genome Atlas (TCGA, https://www.cancer.gov/tcga), and the expression of WIPI2 in colorectal cancer versus normal tissues was analyzed by limma package, and the relationship between clinical traits and WIPI2 expression and prognosis was assessed by univariate and multifactorial cox analysis. The accuracy of the risk model constructed with WIPI2 was also tested by ROC curves as well as survival analysis. The relationship between WIPI2 expression and clinicopathological features was examined using cox analysis. GO and KEGG were performed by cluster Profiler package.

### Immunohistochemistry

The expression of WIPI2, ACSL4 and GPX4 in human colorectal cancer tissues was detected by IHC. Paraffin tissue sections of human colorectal cancer were collected from the Jiangda Diagnostic Pathology Center. These sections were dewaxed in xylene and hydrated in a gradient ethanol concentration, and then used with 3% H_2_O_2_ at room temperature to block endogenous peroxidase activity and incubated with a containment buffer (normal goat serum) at room temperature. The blocking buffer was then discarded, and the PBS washed 3 times. The sections were then incubated overnight at 4°C with purified WIPI2 primary antibody (dilution 1:150; ab105459; Abcam) and incubated with the corresponding secondary antibody and finally stained with DAB and hematoxylin. The IHC sections were further scanned and analyzed respectively with 3DHISTECH and ImageJ.

### Cell lines and cell culture

Human CRC cell lines HCT116 and HT29 were acquired from the China Center for Type Culture Collection (CCTCC) . All cell lines were free from mycoplasma contamination. Cells were cultured in DMEM medium, supplemented with 10% fetal bovine serum (FBS) and 1%Penicillin/Streptomycin. All cells were cultured in 37°C incubator containing 5%CO_2_.

### Small interfering RNA and transfection.

The WIPI2-siRNA sequence (5’-UACGGAAGAUGUGUGCAUUTT-3 5′-CCCUAGCUGUUGGUAGUAATT-3) and the NC-siRNA sequence (5′-UUCUCCGAACGUGUCACGUTT-3) were purchased from GenePharma (Shanghai, China). The si-WIPI2 and si-NC were transfected into HCT116 and HT29 cells, respectively, using Lipofectamine 8000 reagent (Beyotime Biotechnology, Shanghai, China) according to the experimental design and the protocols. Briefly, colon cancer cells at logarithmic growth stage were inoculated in 6-well plates and transfected when the cell confluence reached 70%-80%. Fresh culture medium (complete culture medium containing serum and antibiotics) was changed 20 min before transfection, then a mixture of Lipofectamine 8000 and the above plasmid was prepared using DMEM culture medium without serum and antibiotics according to the instructions, added to the cells in uniform drops, and the cells continued to be cultured for 48-72h before being collected for subsequent experiments.

### Immunofluorescence

The treated cell slides were fixed with 4% paraformaldehyde (Beyotime Biotechnology, Shanghai, China) for 15min, washed 3 times with PBS. 0.5% Triton X-100 was permeabilized at room temperature for 40min, washed 3 times with PBS, 3%BSA was added dropwise, and closed at room temperature for 1h. The WIPI2 primary antibody and fluorescent secondary antibody were added dropwise according to the experimental requirements, and finally the cell nucleus were stained and sealed with antifade mounting medium Hoechst (Beyotime Biotechnology, Shanghai, China), and then the images were collected under the fluorescence microscope.

### Cell treatment

HCT116 and HT29 cells were treated with different concentrations of Erastin (0, 10, 20, and 40 μM) for 24 h, and then viability of the cells was assayed. After transfection with si-RNAs or si-NC, suitable CRC cells were treated with Erastin (10 μM) for 24 h as needed.

### CCK-8 assay

Human colorectal cancer cell lines (6x10^3^/mL) were inoculated into 96-well plates and treated with si-RNAs and si-NC for transfection. The cells were cultured at 37°C for several days according to the experimental requirements. Finally, 10µl of CCK-8 solution (Solarbio, Beijing, China) was added to each well, then the cells were continued incubated in 37˚C incubator for 2-3h, and finally the absorbance of each well was evaluated at 450 nm using an enzyme marker. All CCK-8 experiments were repeated at least three times.

### Colony formation assay

HCT116 and HT29 human colorectal cancer cells (2×10^3^/ml) were inoculated into 6-well culture plates at per well and they were treated with si-RNA and si-NC transfection for 7~14 days. The medium was discarded, and the cells were fixed with 4% paraformaldehyde for 10min and then stained with crystal violet solution (Beyotime Biotechnology, Shanghai, China) for 10min. After washing three times with PBS, the colonies were imaged and quantified using a camera.

### Western blot

First, colorectal cancer cells were treated by lysis at 4°C using RIPA lysis solution (Beyotime Biotechnology, Shanghai, China) containing fresh protease and phosphatase inhibitors, and 12,000g lysate was collected. After being freezing centrifuged for 20 minutes, the supernatant was removed to collect total cellular proteins. Antibodies used for Western blot were WIPI2 (1:1000), ACSL4 (1:5000) and GPX4 (1:1000), all purchased from Abcam, USA, and β-actin (1:1000), purchased from Beyotime Biotechnology, Shanghai, China. Thereafter, PVDF membranes were incubated with peroxidase-conjugated secondary antibody (1:1000, Beyotime Biotechnology) at 37°C for 1h. Finally, the signal was visualized using Enhanced chemiluminescence and the images were analyzed using ImageJ analysis software. All experiments were repeated at least 3 times.

### Statistical analysis

All data were statistically analyzed using Graphpad prism software (Graphpad Prism6). Experimental data was expressed as mean ± standard deviation (SD). Differences between groups were compared using t-test, one-way or two-way analysis of variance (ANOVA). p < 0.05 was considered a statistically significant difference.

## Results

### Screening for ferroptosis genes

**Figure 1:**
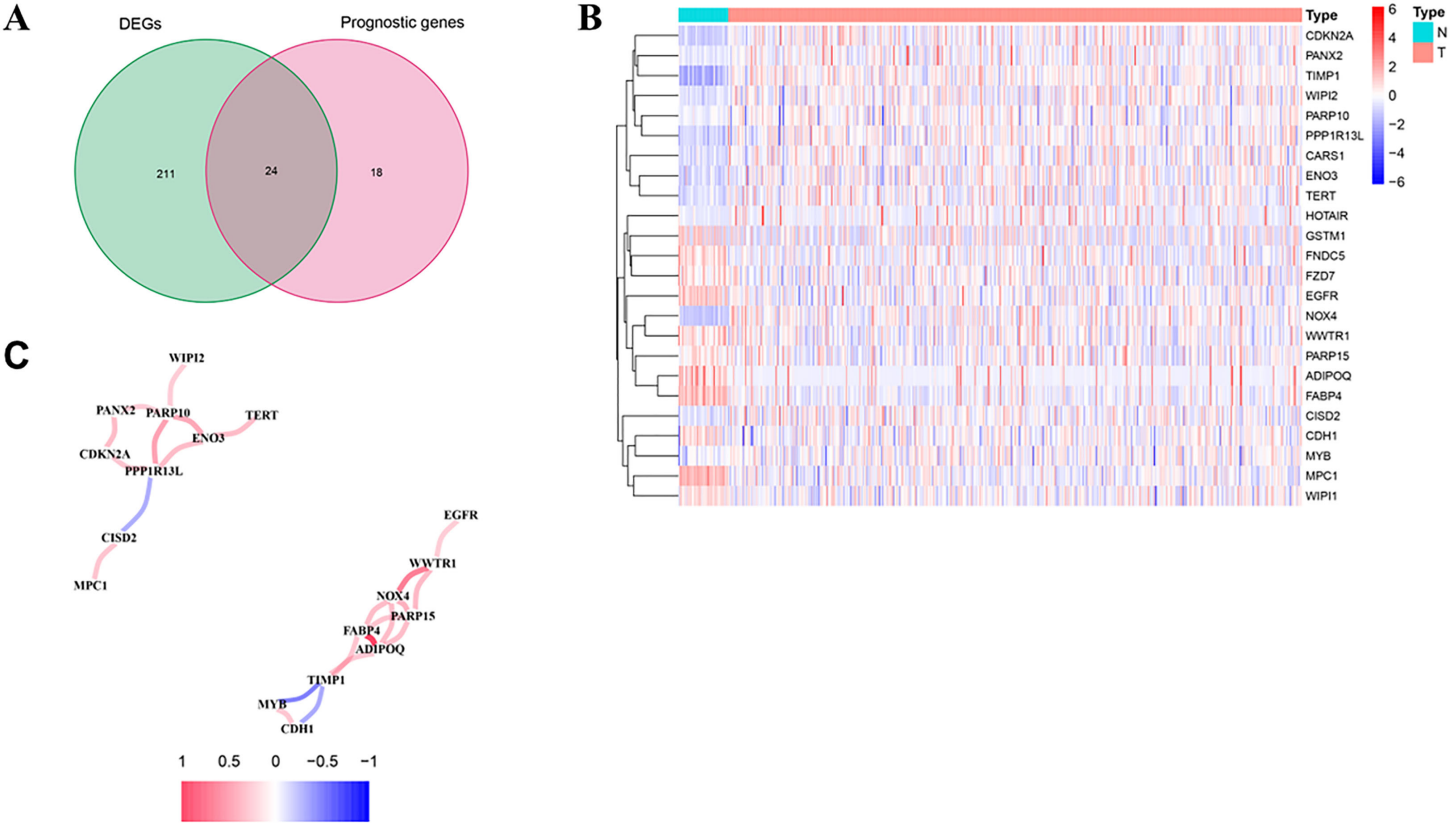
Screening for ferroptosis genes associated with prognosis in colorectal cancer **(A)** TCGA collection of ferroptosis differentially expressed genes and associated prognostic genes. **(B)** Heat map visualizing the expression of 24 genes in colorectal cancer tissues. **(C)** The interrelationships among the 24 genes.

We first analyzed the TCGA database for differentially expressed ferroptosis genes and associated prognostic genes and screened 24 genes by taking their intersection (**Figure 1A**). We further visualized the expression of these 24 genes in the tissues of colon adenocarcinoma patients by heat map (**Figure 1B**) and constructed a protein-protein interaction network to observe the correlation between them (**Figure 1C**). The risk values (Hazard ratio, HR) of these 24 genes were further obtained by Cox proportional risk regression models based on clinical traits, and thirteen high-risk genes (IFNA1, MIR9-3HG, BRDT, LINC00336, ATG13, ALOX12, FNDC5, PHF21A, ENO3, TERT, PTPN6, CARS1和WIPI2) had HR>1.9, all of which were associated with poor prognosis (**Table S1**). After further review of relevant research reports, we finally selected WIPI2 as the target gene for in-depth study.

### The expression of WIPI2 in human colorectal cancer tissues

**Figure 2:**
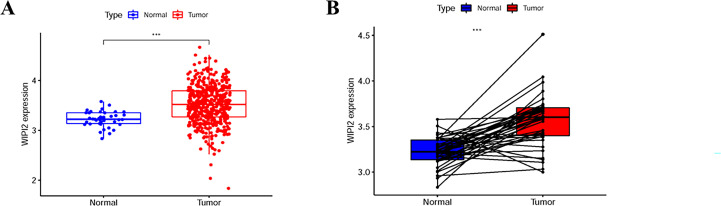
Expression levels of WIPI2 in colorectal cancer patients in TCGA database. **(A)** Scatter plot evaluation of WIPI2 expression in human colorectal cancer tissues. **(B)** Paired plots analyzing the expression of WIPI2 in human colorectal cancer tissues. ***P < 0.001.

To further explore the importance of WIPI2 in colorectal cancer, we collected 41 normal tissues and 473 colorectal cancer tissues from TCGA. What’s more, we visualized the differential expression of WIPI2 in TCGA-CRC patients (**Figure 2A**), and it was seen that the expression level of WIPI2 was greatly elevated in CRC patients (p<0.05). We further investigated the expression levels of WIPI2 in tumor tissue and its paired paracancerous normal tissues in TCGA-CRC patients (**Figure 2B**), and both results suggested that the expression of WIPI2 was higher in colorectal cancer tissues compared to normal tissue with notable differences (p<0.05).

### Prognostic relevance of WIPI2 in patients with colorectal cancer

**Figure 3:**
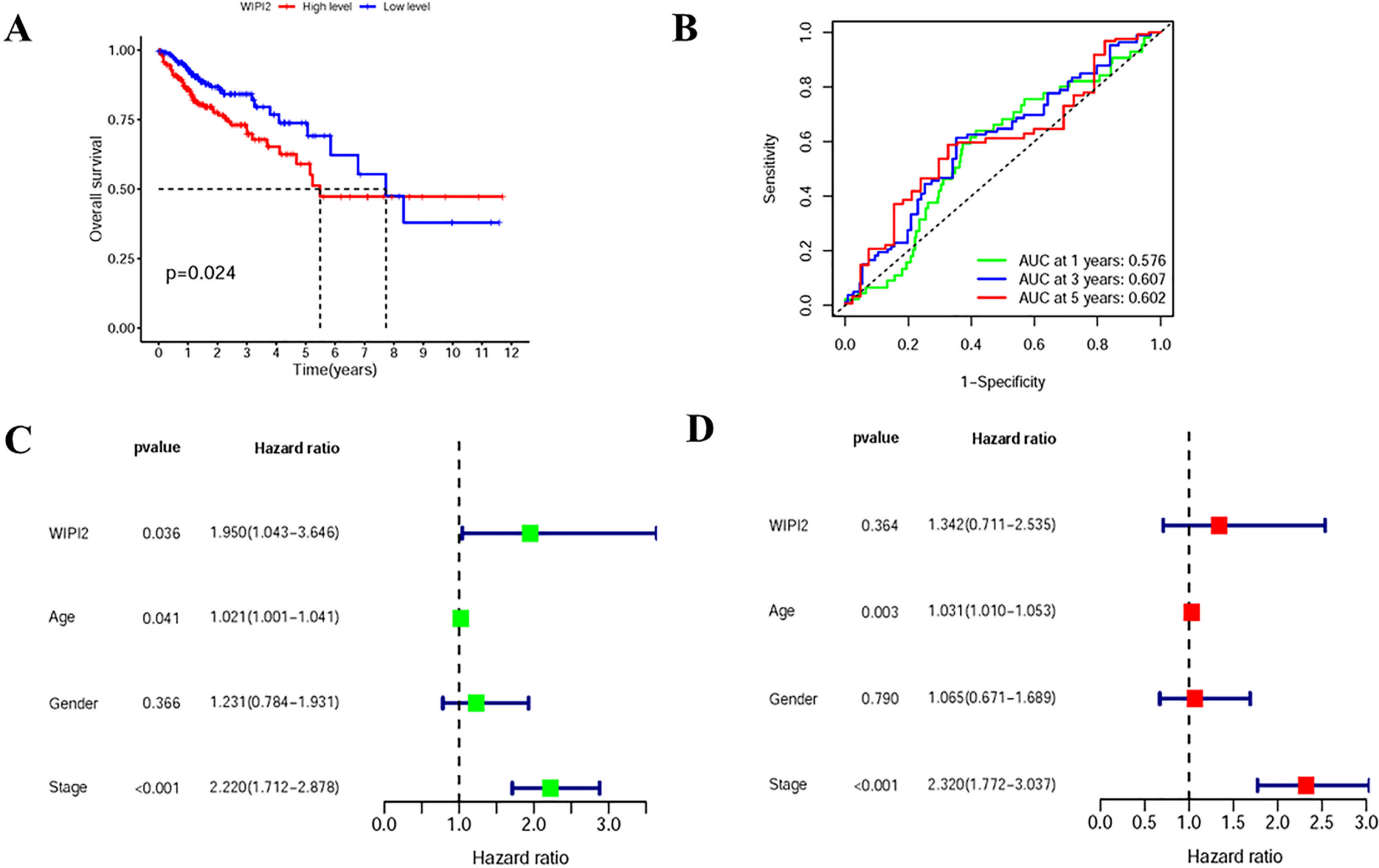
Prognostic analysis of survival of WIPI2 in colorectal cancer patients **(A)** Survival curves for different WIPI2 expression levels. **(B)** ROC curve analysis to compare the five-year survival of patients. **(C)** One-way COX analysis to assess the effect of WIPI2, age, gender and stage on patient prognosis. **(D)** Multi-factor COX analysis to assess the effect of WIPI2, age, gender and stage on patient prognosis.

To assess the relationship between WIPI2 and prognosis, we divided CRC patients into high and low expression groups based on the median expression of WIPI2 and found that the overall survival of the high WIPI2 expression group was lower than that of the lower group (p<0.05) (**Figure 3A**). The accuracy of WIPI2 on disease prognosis was assessed by ROC curves comparing patients’ five-year survival rates and calculating the area under curve (AUC), which was found to be >0.5 for all 5-year risk prognostic assessment models (**Figure 3B**). To further investigate the role of WIPI2 in colorectal cancer, a univariate independent prognostic analysis revealed that WIPI2 was significant with age and stage, but not with gender (**Figure 3C**). Further by multi-factor independent prognostic analysis, only age and stage were found significantly different (**Figure 3D**), suggesting that WIPI2 may not yet be an independent prognostic factor for effectively predicting patient prognosis. The above results suggest that the relatively high expression of WIPI2 may predict the advanced colorectal cancer with poor prognosis.

### Relationship between WIPI2 expression and clinicopathological features

**Figure 4:**
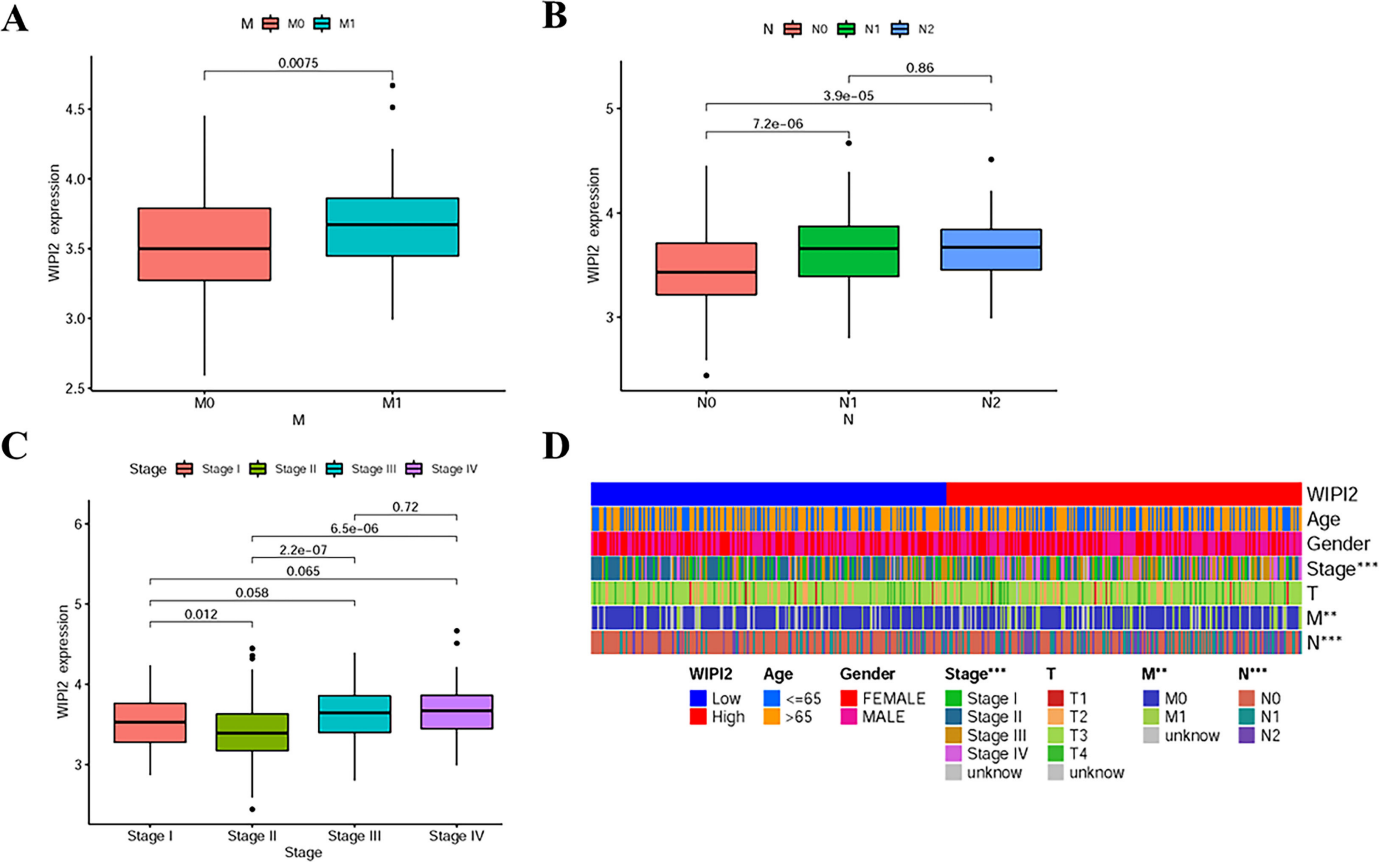
Relationship between WIPI2 and clinicopathological features **(A)** WIPI2 expression in M-stage. **(B)** WIPI2 expression in N-stage. **(C)**WIPI2 expression in clinical stage. **(D)** Heat map analysis of differential expression of WIPI2. **P < 0.01, ***P < 0.001.

Next, the association between the expression level of WIPI2 and prognosis-related clinicopathological characteristics, such as whether patients had distant metastases (M), whether they had regional lymph node metastases (N), clinical stage and depth of tumor infiltration (T), was further verified. The results showed that WIPI2 expression gradually increased with the progression of the disease in M1 stage, N stage and decreased in clinical stage II (**Figures 4A-C**). The heat map analysis indeed observed that differential expression of WIPI2 is significantly different from these three clinical stages (**Figure 4D**), indicating that WIPI2 is a gene that is associated with multiple clinical stages. In addition to bioinformatics analysis, we also found that the expression of WIPI2 correlated with the degree of tumor differentiation and age of patients by sorting out the pathological information collected from colorectal patients (**Table S2** and **Figure S1**), which also indicates that WIPI2 is indeed a gene associated with multiple clinical pathological features.

### The expression of WIPI2 is higher in colorectal cancer tissues than in paracancerous tissues

**Figure 5:**
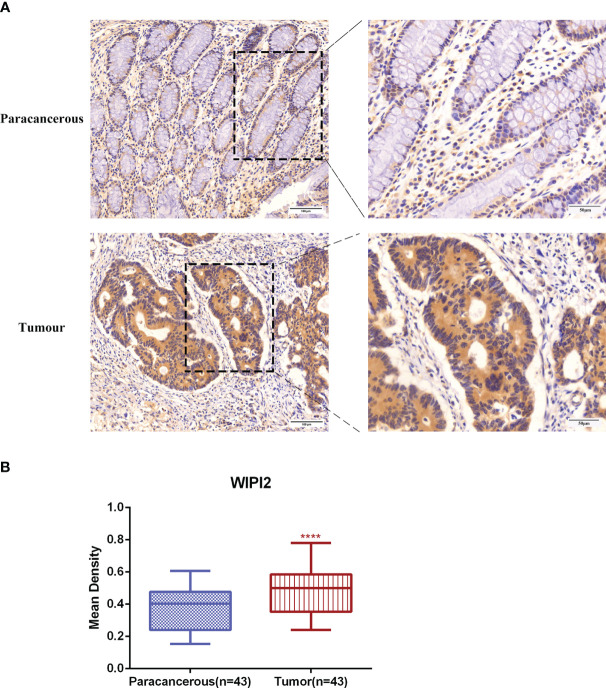
Immunohistochemical examination of WIPI2 expression in human colorectal cancer tissues. **(A)** Immunohistochemical staining of WIPI2(n=43) in colorectal carcinoma tissues and adjacent normal tissues. Scale bar, 100 µm, 50µm. **(B)** Mean optical density IOD/AREA (Mean Density) of WIPI2 in colorectal carcinoma tissues and corresponding adjacent normal tissues, ****P < 0.001.

To evaluate the expression level of WIPI2 in colorectal cancer tissues, we explored the expression of WIPI2 in CRC tissues and paracancerous tissues by immunohistochemical staining. IHC results showed that WIPI2 was weakly positive expressed in the cytoplasm in paracancerous colorectal tissues and moderately to strongly positive expressed in the cytoplasm of tumor cells in tumor tissues (**Figure 5A**). In conclusion, the expression intensity degree of WIPI2 was higher in most colorectal cancer tissues than in paracellular tissues (**Figure 5B**).

### Downregulation of WIPI2 inhibits the growth and proliferation of CRC cells

**Figure 6:**
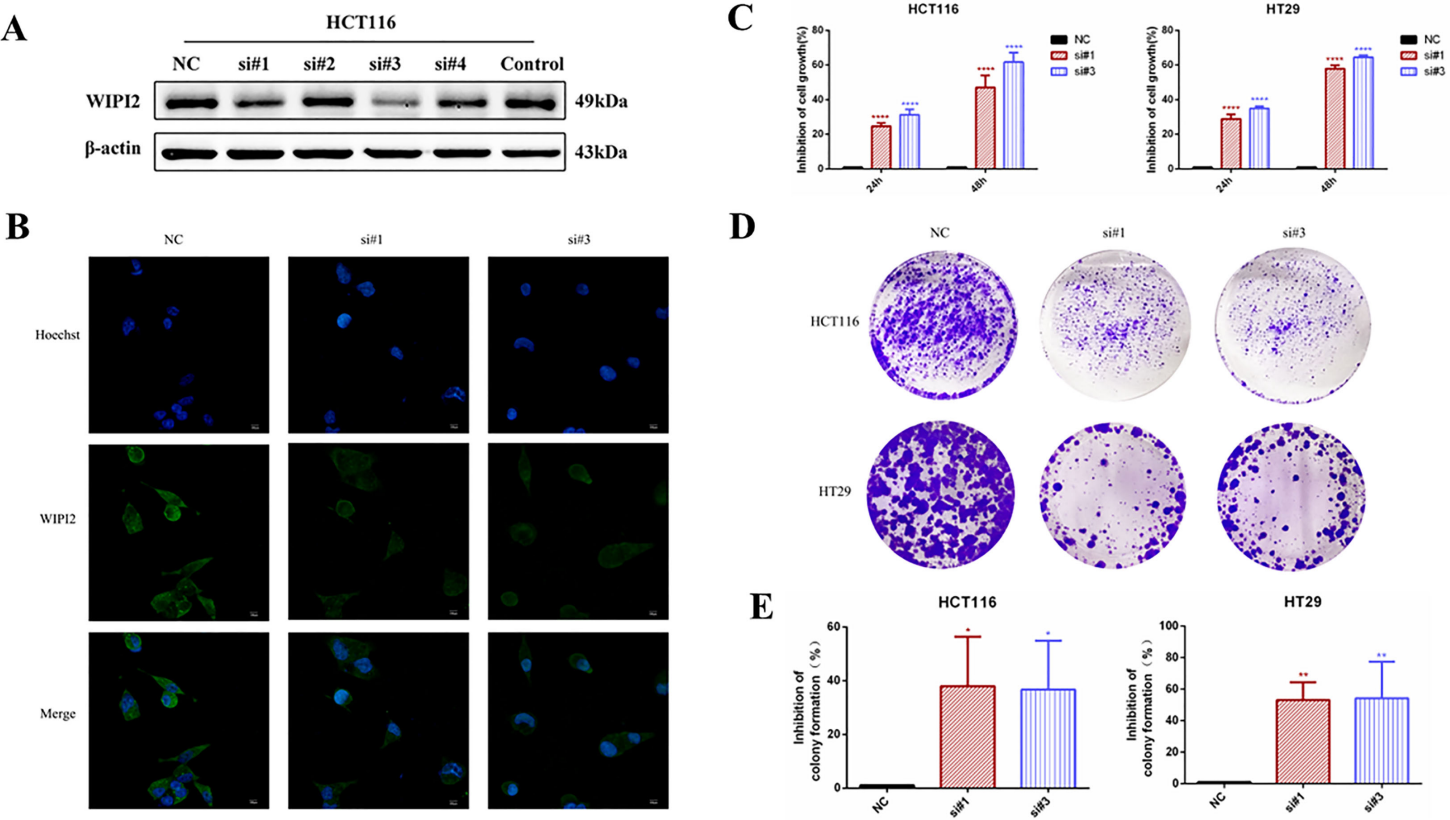
Effects of knockdown of WIPI2 on the growth and proliferation of colorectal cancer cells. **(A)** Expression of four si-RNAs targeting WIPI2 in HCT116 was detected by Western blot. **(B)** Immunofluorescence detection of fluorescence expression intensity of transfected si-NC and si-WIPI2 in HCT116 cells. Scale bar, 100 µm. **(C)** CCK-8 assay to detect cell growth activity after transfection with si-NC and si-WIPI2. **(D)** Cell clone formation assay to detect cell proliferation after transfection with si-NC and si-WIPI2. **(E)** The statistics of colony formation. *P < 0.05, **P < 0.01, ****P < 0.0001.

In this research, two si-RNAs targeting WIPI2, si#1 and si#3 were selected to inhibit the expression of WIPI2 in colorectal cancer cell lines (**Figure 6A**) to validate its regulatory role in colorectal cancer cells *in vitro*. After transient transfection of human colorectal cancer HCT116 cells with si-RNA, the protein expression level and fluorescence intensity of WIPI2 were reduced (**Figure 6B**).

To find out the function of WIPI2 on the proliferation of colorectal cancer cells, its cellular activity was tested using CCK-8 and cell colony formation assays after down-regulation of WIPI2. CCK-8 results showed that depletion of WIPI2 (si-WIPI2) substantially inhibited the growth activity of colorectal cancer cells in the si-groups compared to the NC-group, and the inhibition rate was more significant in the 48h group compared to the 24h group (**Figure 6C**). Moreover, the results of the cell colony formation assay also showed that cell proliferation was also notably reduced in the si-groups after knockdown of WIPI2 in comparison to the NC-group (**Figures 6D, E**).

### WIPI2 induces changes in the expression of ferroptosis proteins

**Figure 7:**
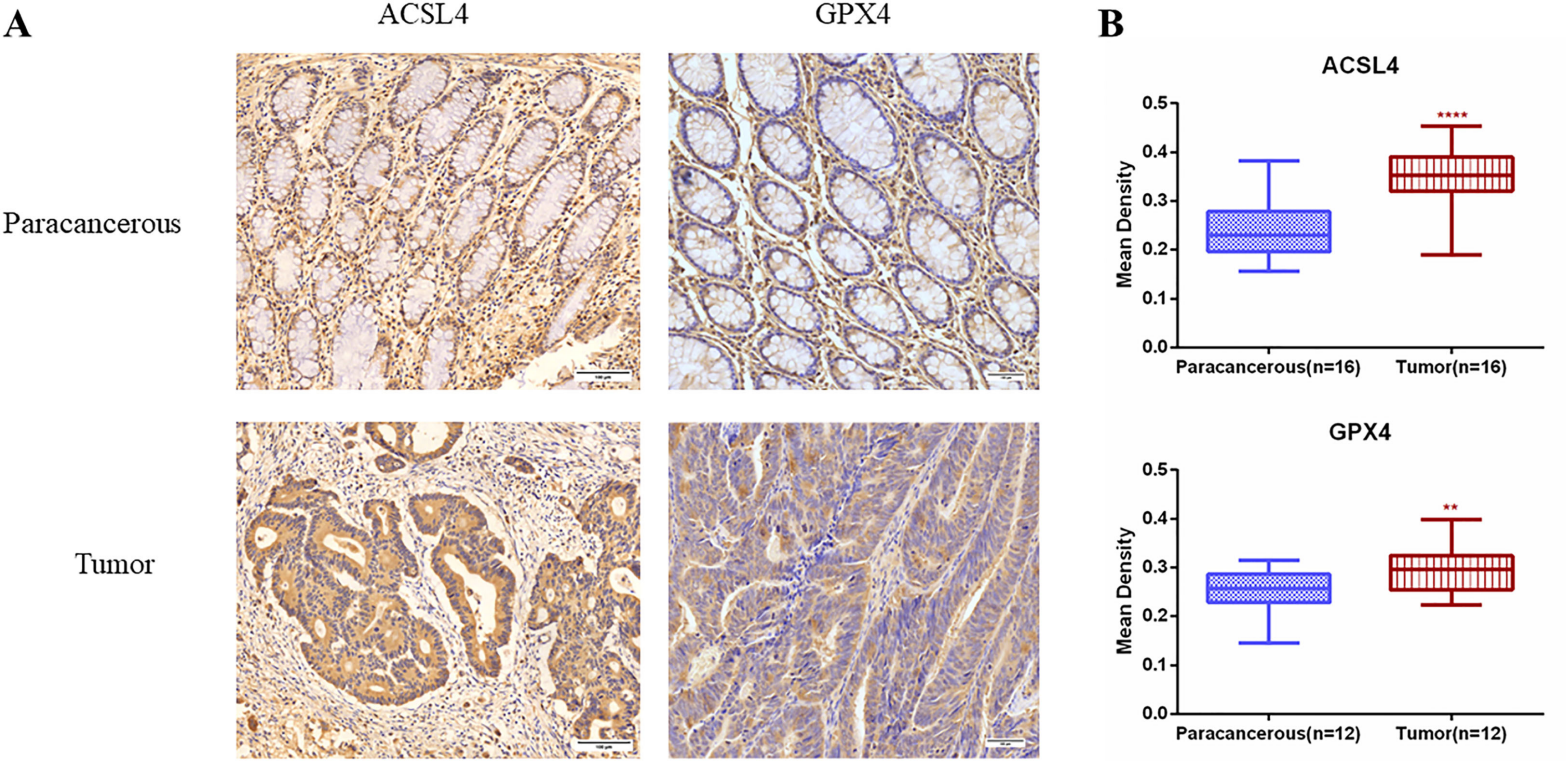
Immunohistochemical examination of ACSL4 and GPX4 expression in human colorectal cancer tissues. **(A)** Immunohistochemical staining of ACSL4(n=16) and GPX4(n=12) in colorectal carcinoma tissues and adjacent normal tissues. Scale bar, 100 µm. **(B)** Mean optical density IOD/AREA (Mean Density) of ACSL4 and GPX4 in colorectal carcinoma tissues and corresponding adjacent normal tissues, statistical analysis **P < 0.01, ****P < 0.0001.

**Figure 8:**
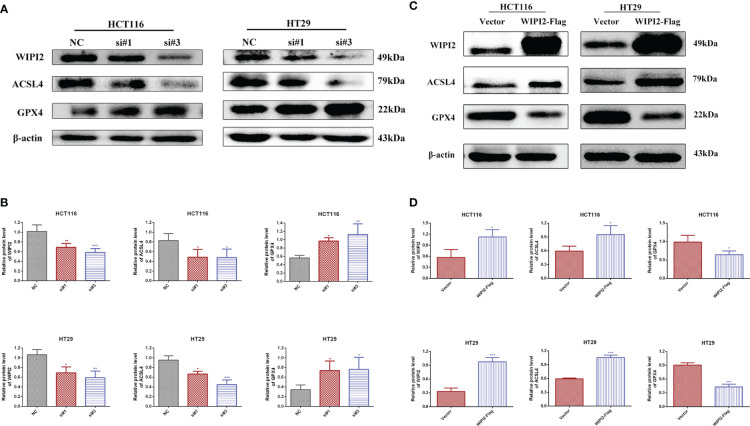
Changes in ferroptosis proteins after treatments of WIPI2. **(A)** Changes in ferroptosis protein expression after knockdown of WIPI2 detected by Western blot. **(B)** Statistical analysis of proteins in HCT116 and HT29 cells. **(C)** Changes in ferroptosis protein expression after overexpression of WIPI2 detected by Western blot. **(D)** Statistical analysis of proteins in HCT116 and HT29 cells. *P < 0.05, **P < 0.01, ***P < 0.001.

We preliminary searched in the FerrDb database found that WIPI2 may be a potential positive regulator of ferroptosis, so we wanted to further explore whether the effect of WIPI2 on colorectal cancer cell activity is associated with ferroptosis. Studies have reported that acyl coenzyme A (CoA) synthase long chain family member 4 (ACSL4) is involved in the synthesis of easily oxidized membrane phospholipids such as phosphatidylethanolamine or phosphatidylinositol, which promote lipid peroxidation of polyunsaturated fatty acids and are thus involved in ferroptosis occurrence (17). The lipid hydroperoxidase GPX4 transforms lipid hydroperoxides to lipid alcohols and prevents the formation of toxic lipid reactive oxygen species caused by iron ions (Fe^2+^). The suppression of GPX4 activity leads to the formation of lipid ROS and lipid peroxidation, which induces ferroptosis (18, 19). Moreover, our preliminary immunohistochemical staining showed that the expression intensity of both ACSL4 and GPX4 was higher in colorectal cancer tissues than in paracancerous tissues (**Figures 7A, B**), which also suggested that ferroptosis might have a regulatory role in colorectal cancer. Furthermore, we want to investigate the correlation between WIPI2 and ferroptosis in colorectal cancer cells, the results of Western blot showed that WIPI2 knockdown (si-WIPI2) significantly inhibited the expression of ferroptosis protein ACSL4 and increased the expression of GPX4 compared with the NC-group (**Figures 8A, B**). Similarly, when WIPI2 was overexpressed, the expression of ACSL4 and GPX4 was reversed (**Figures 8C, D**). The results tentatively suggest that there may be a positive correlation between WIPI2 and ferroptosis.

### Downregulation of WIPI2 reduces the sensitivity of CRC cells to Erastin

**Figure 9:**
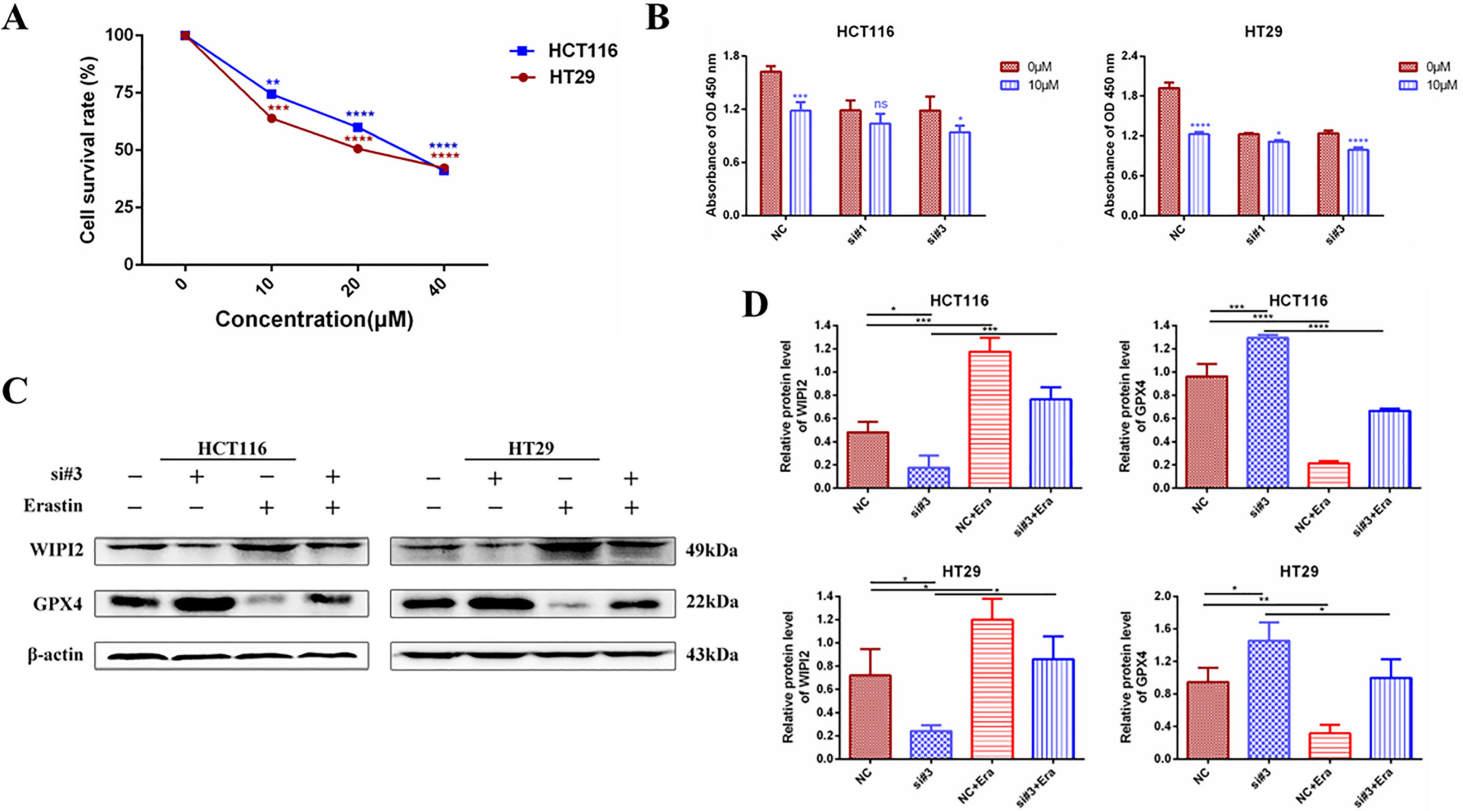
Changes in cellular activity and ferroptosis protein expression after combined treatment with knockdown of WIPI2 and Erastin. **(A)** Cellular activity assay after treatment of CRC cells with different concentrations of Erastin compared to 0μM (DMSO 0.1%). **(B)** Cellular viability assay after knockdown of WIPI2 and Erastin combined with cell treatment. **(C)** Changes in ferroptosis protein expression after knockdown of WIPI2 detected by Western blot. **(D)**Statistical analysis of proteins in HCT116 and HT29 cells. *P < 0.05, **P < 0.01, ***P < 0.001, ****P < 0.0001, ns: no significance.

It was found that (20), Erastin, one of the classical ferroptosis inducers, has significant cytotoxic effects on colorectal cancer cells. Erastin inhibits the activity of the cysteine-glutamate reverse transporter (System Xc^-^) to reduce the expression level of GPX4, leading to a reduction in cellular antioxidant capacity and the accumulation of ROS, ultimately causing lipid peroxidative cell death (21). Next, we further investigated the sensitivity of WIPI2 to the ferroptosis inducer Erastin in colorectal cancer cells. Firstly, we verified whether the ferroptosis inducer Erastin could induce death in CRC cells. After treating HCT116 and HT29 cells with different concentrations of Erastin (0, 10, 20 and 40 μM) for 24 h, CCK-8 measured the cellular activity and showed that Erastin had a concentration-dependent growth inhibitory effect on both cell lines (**Figure 9A**). Next, we used Erastin and transfected si-WIPI2 to detect cellular viability after combined treatment of colorectal cancer cells with CCK-8. The results showed that cell viability was more inhibited in both the NC-10μM group and the si-10μM group after Erastin treatment. However, the inhibition rate in the NC-10μM group was significantly higher than that in the si-10μM groups compared to the 0μM group (**Figure 9B**). This initially suggested that WIPI2 can further enhance the inhibitory effect of Erastin on cellular activity. Subsequently, we wanted to further verify whether Erastin could induce changes of protein level. As shown in **Figure 9C**, treatment of Erastin (10μM) was able to induce an increase in WIPI2 expression and a decrease in ferroptosis protein GPX4 expression. However, the trends of protein level changes in the NC-group were also more significantly than in the si-groups. These results suggested that WIPI2 could enhance the susceptibility of colorectal cancer cells to Erastin by regulating the expression activity of GPX4.

## Discussion

Colorectal cancer, as one of the common malignant tumors, has the highest incidence and mortality rate in China and has severely endangered the health of the human population (22). Therefore, in order to improve the current situation of colorectal cancer patients, it is necessary and critical to identify new indicators for prognostic assessment and targeted therapy of colorectal cancer.

Ferroptosis is a new form of regulated cell death characterized by lipid peroxide- and iron-dependent hyperaccumulation (23). Recent researches have revealed that ferroptosis plays a major role in numerous human diseases such as Huntington’s chorea, neoplastic diseases, acute kidney injury and ischemia-reperfusion injury (24, 25). Ferroptosis may also be used in cancer therapy by inhibiting tumor cell growth (18). It has been reported that in colorectal cancer, cetuximab can stimulate ferroptosis in KRAS mutant colorectal cancer cells by inhibiting the NFF2/HO-1 signaling axis, thereby increasing their cytotoxicity (26); Lipocalin-2 (27) and SRSF9 (28) can promote the activity of ferroptosis proteins SLC7A11, GPX4 to inhibit ferroptosis in colon cancer cells, and thereby affecting cellular drug resistance. This suggests that ferroptosis is essential to the development, progression and treatment in colorectal cancer. In addition, some ferroptosis regulators have also shown promise as highly effective and sensitive diagnostic and prognostic biomarkers in cancer patients (29). Therefore, screening for ferroptosis-associated biomarkers in colorectal cancer can provide potential targets for clinical prediction of recurrence, metastasis and prognosis of colorectal cancer, making ferroptosis a more effective strategy for tumor treatment.

In this study, 24 differential ferroptosis-related genes were screened by bioinformatics analysis in the early stage, and further by Cox analysis of these genes with risk values, 13 high-risk genes were obtained, and WIPI2 was finally selected for follow-up study by further comparison. Gene expression profiling data showed that WIPI2 was significantly more expressed in colorectal cancer tissues than in normal tissues, and this result was affirmed by immunohistochemical section staining results. Independent univariate and multivariate prognostic analyses revealed significant differences between WIPI2 and colorectal cancer patients in terms of gender, age and distant metastasis (M stage), metastasis of regional lymph node (N stage) and clinical stage II, with WIPI2 expression increasing as patients’ disease worsened. Furthermore, we found that the expression of WIPI2 correlated with the degree of tumor differentiation and age of patients by sorting out the pathological information collected from colorectal patients, which also indicates that WIPI2 is indeed a gene associated with multiple pathological features. However, WIPI2 still cannot be used as an independent prognostic factor for colorectal adenocarcinoma at this time. A comparison of five-year survival rates as well as overall survival curves revealed significant differences between high and low risk groups, with higher levels of WIPI2 expression often predicting shorter overall survival, suggesting that the risk model can be a good differentiator of patient survival. All the above bioinformatics analysis results suggest that WIPI2 is a potential ferroptosis differential expression gene and prognostic gene in colorectal cancer, which may have an influence in the development and progression of CRC.

It has been demonstrated in earlier studies that the human WIPI gene is aberrantly expressed in many cancer types (13). For example, WIPI2 and WIPI4 mRNAs are significantly differentially expressed in advanced pancreatic cancer, and WIPI-mediated autophagic activity is progressively reduced in precancerous stages during the transformation of pancreatic tumors to adenocarcinoma, suggesting that aberrant expression and altered activity of WIPI genes have important implications in the development of various cancers (13). In addition, WIPIs family proteins are thought to have a very important relationship with autophagy, serving as effectors of autophagy-specific phosphatidylinositol-3-phosphate (Ptdlns3P) and playing an important role in both recognition and decoding of Ptdlns3P signaling in de nascent autophagy *in vivo (* 30). And it also serves as a scaffold to recruit other proteins or protein complexes that contribute to the nucleation and expansion of autophagosomal membranes (31). Mutations in these proteins can severely affect the autophagic process and are strongly related with various cancers and diseases such as neuronal degeneration and intellectual disability (32).

WIPI2 is a member of the WIPI family and the mammalian homolog of the yeast gene ATG18. This autophagy-associated gene is a key protein in isolation membrane genesis and elongation and is thought to be a key downstream substrate of m TOR-regulated autophagy, with a very important role in the autophagic process (16). WIPI2 is crucial for LC3 lipidation and is able to bind specifically to ATG16L1, thereby recruiting the ATG12-ATG5-ATG16L1 complex required for LC3 lipidation, which can further link the PI3KC3-mediated Ptd Ins3P products and LC3 lipidation during phagocytic vesicle formation and expansion (33). Currently, most studies have focused on the impact and role of WIPI2 in autophagy, but less on its role in other pathways. Therefore, our study is dedicated to research the potential cancer-promoting mechanisms of WIPI2 in CRC, with a view to filling some of the gaps in WIPI2 research and bringing new strategies and ideas to the diagnosis and treatment of CRC.

Regarding the pro-tumor effects of WIPI2, a recent research found that the expression of WIPI2 was higher in human hepatocellular carcinoma tissues and its high expression predicted a poor prognosis for HCC patients. In addition, WIPI2 regulates the growth of hepatocellular carcinoma cells mainly through the AMPK/AKT/Cyclin D1 pathway and causes apoptosis via caspase-3 and Bcl2 (16). Meanwhile, we also proved for the first time the relationship between WIPI2 expression and colorectal cancer cell growth. We discovered that the expression of WIPI2 was greatly increased in human colorectal cancer tissues compared to paracancerous tissues. *In vitro* cellular assays also showed that when the expression of WIPI2 was knocked down, the growth and proliferation of colorectal cancer cells could be significantly inhibited.

ACSL4 is a protein of the long chain lipoyl CoA synthetase (ACSL) family and has been found as a key gene and essential molecule in the iron death pathway. ACSL4 synthesizes arachidonic acid and adrenoic acid into arachidonic CoA and adrenoyl CoA, respectively, to participate in membrane phospholipid synthesis. Further treatment with ferroptosis inducers such as RSL3 resulted in the oxidation of long-chain polyunsaturated fatty acids in the membrane, which triggered cellular ferroptosis (34). Meanwhile, other studies have shown that GPX4, a key enzyme for intracellular glutathione synthesis, is significantly over expressed in 13 cancers (35). It is also able to trigger ferroptosis by inhibiting GPX4 expression *in vitro* and in mouse xenograft models (36). In colorectal cancer cells, RSL-3 induced the onset of ferroptosis by prompting GPX4 inactivation, thereby reducing the proliferative capacity of tumor cells (37). In kidney and ovarian clear cell carcinoma, inhibition of GPX4 expression also promoted the development of ferroptosis (38). Our study found that knockdown of WIPI2 expression (si-WIPI2) inhibited ACSL4 expression and elevated GPX4 expression compared to the NC-group. When overexpressed WIPI2, this change could be reversed. The results all suggest that WIPI2 may influence the ferroptosis pathway in the development of colorectal cancer by regulating the activity of ferroptosis proteins.

Erastin, a classical inducer of ferroptosis, was the first compound identified in a phenotypic screen to be selectively lethal to tumor cells, and has been shown in several tumor studies. and has shown efficacy in several tumor studies (39). It has been demonstrated that Erastin can inhibit the proliferation and progression of HCC by inducing ferroptosis, and thus has anti-tumor effects. In mouse tumor models, Erastin has also been shown to inhibit tumor growth (40). Therefore, Erastin is most likely to be considered as a potential drug for cancer treatment, which also provides new targets and ideas for the treatment of tumors as well.GPX4, as one of the targets of the ferroptosis inducer Erastin, can function by catalyzing the reduction of lipid peroxides. A diminish in GPX4 activity and an increase in lipid peroxidation levels are both key factors in the development of ferroptosis (39). Our study also confirmed that after Erastin induced ferroptosis in colorectal cancer cells, the expression of WIPI2 at the protein level was significantly increased compared to both cells without induced ferroptosis, which verified our conjecture that WIPI2 is involved in Erastin-induced ferroptosis process in colorectal cancer cells. And WIPI2 can further enhance the inhibitory effect of Erastin on cell viability and protein expression changes. This suggests that WIPI2 may influence the ferroptosis induced by Erastin in CRC cells by regulating the expression of GPX4. At the same time, there are still many limitations in this study, such as the mechanism of WIPI2 and ferroptosis is still relatively superficial, but the key regulatory molecules of ferroptosis such as GPX4 and ACSL4 have been identified to have an influence in this research, while more in-depth mechanisms and new targets with clinical translational value need to be further explored in greater depth.

**Figure 10:**
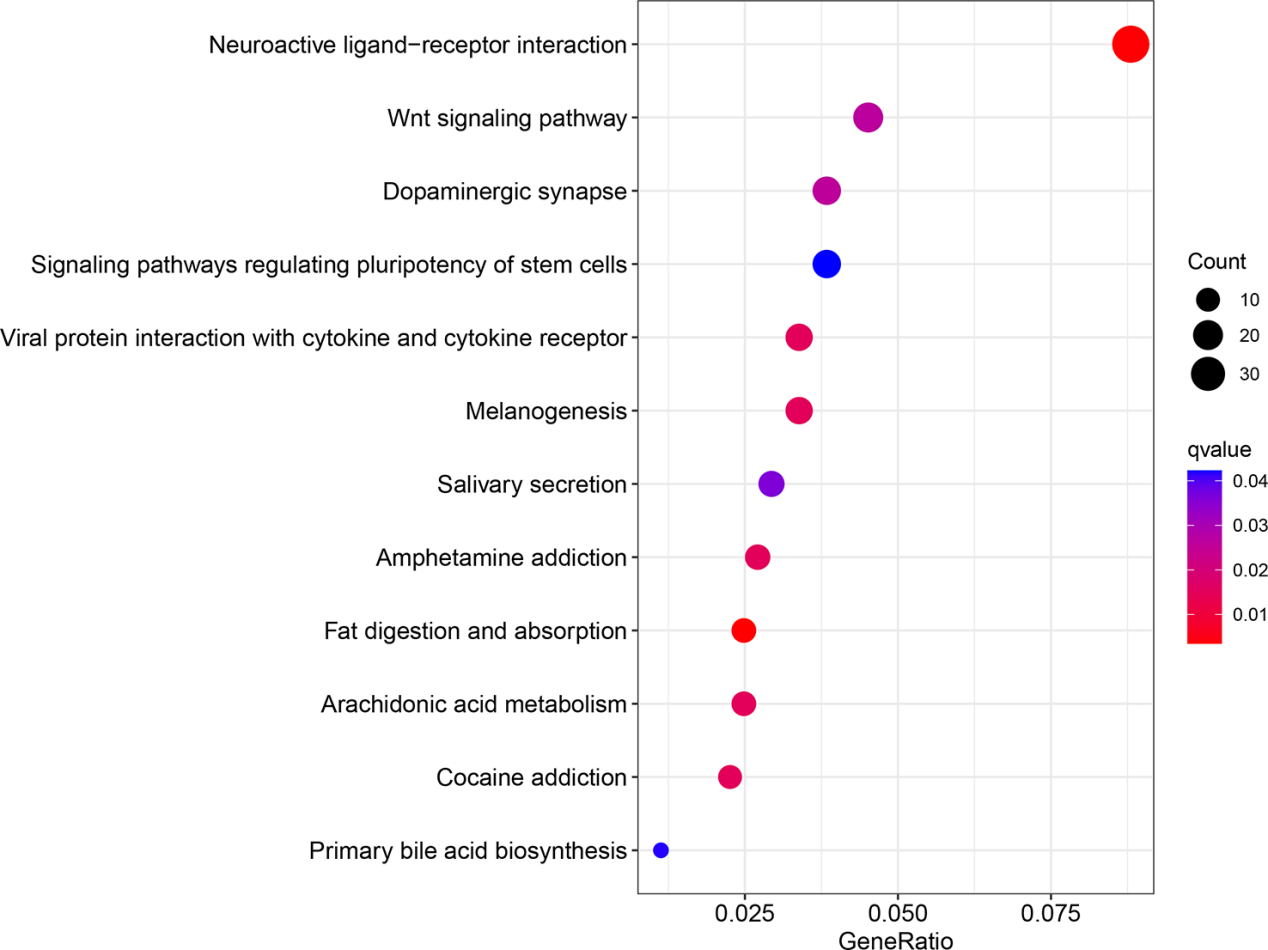
GO enrichment analysis of differential genes.

In summary, our findings showed increased expression of WIPI2 in colorectal cancer tissues compared to adjacent normal tissues, and we will further increase the number of cases so that we can further observe and count whether its high expression predicts a poor prognosis for colorectal cancer patients. In the meantime, we believe that WIPI2 exerts a pro-oncogenic effect in CRC and is able to promote the proliferation of CRC cells. In addition, WIPI2 was able to influence the sensitivity of CRC cells to the ferroptosis inducer Erastin by regulating the protein level of GPX4. Therefore, WIPI2 is most likely one of the important regulatory factors of Erastin-induced ferroptosis in human colorectal cancer cells. Furthermore, the pro-tumor effect of WIPI2 and its correlation with ferroptosis suggest that there may be other more dominant mechanisms, such as autophagy, for the regulatory role of WIPI2 on tumor cells. Furthermore, as shown in our analysis of the GO pathway enriched for high and low levels of WIPI2 respectively, WIPI2 was also significantly associated with the regulation of stem cell diversity (**Figure 10**), suggesting that WIPI2 may also exert its regulatory role by regulating the stemness of tumor cells. Also unfortunately, this study has not yet been validated in animal models, and these are subject to further studies to follow.

## Data availability statement

The datasets presented in this study can be found in online repositories. The names of the repository/repositories and accession number(s) can be found in the article/**Supplementary Material**.

## Ethics statement

The studies involving human participants were reviewed and approved by IRB, School of medicine, Jianghan University. Written informed consent for participation was not required for this study in accordance with the national legislation and the institutional requirements.

## Author contributions

LY: design and investigation of the experiment, data compilation, and writing of the first draft. YL: same contribution as the first author. XD: analyzing data and making graphs. MT, HG, RZ, and MC: analysis and organization of data. YCL: experimental guidance. QC: preparation of experimental reagents and materials. YO: clinical case review. HZ: design and guidance of experiments, review and revision of the first draft, and financial support for publication. XW: conceptualization and experimental design of the project, and review and revision of the first draft. All authors contributed to the article and approved the submitted version.

## Supplementary material

Supplementary Figure 1 Relationship between WIPI2 and clinicopathological features **(A)** Relationship between the relative expression levels of WIPI2 and the degree of differentiation of colorectal cancer patients. **(B)** Relationship between the relative expression levels of WIPI2 and the age of colorectal cancer patients. **P < 0.01.

Supplementary Table 1 One-way COX regression analysis of ferroptosis-associated genes.

Supplementary Table 2 Correlation between clinical pathological features and the expression of WIPI2 in colorectal cancer patients.
