# Supplementary figures and images for: WIPI2 enhances the vulnerability of colorectal cancer cells to erastin via bioinformatics analysis and experimental verification
Source: Front Oncol. 2023 May 3;13:1146617. doi: 10.3389/fonc.2023.1146617 (PMC10189881; doi:10.3389/fonc.2023.1146617)

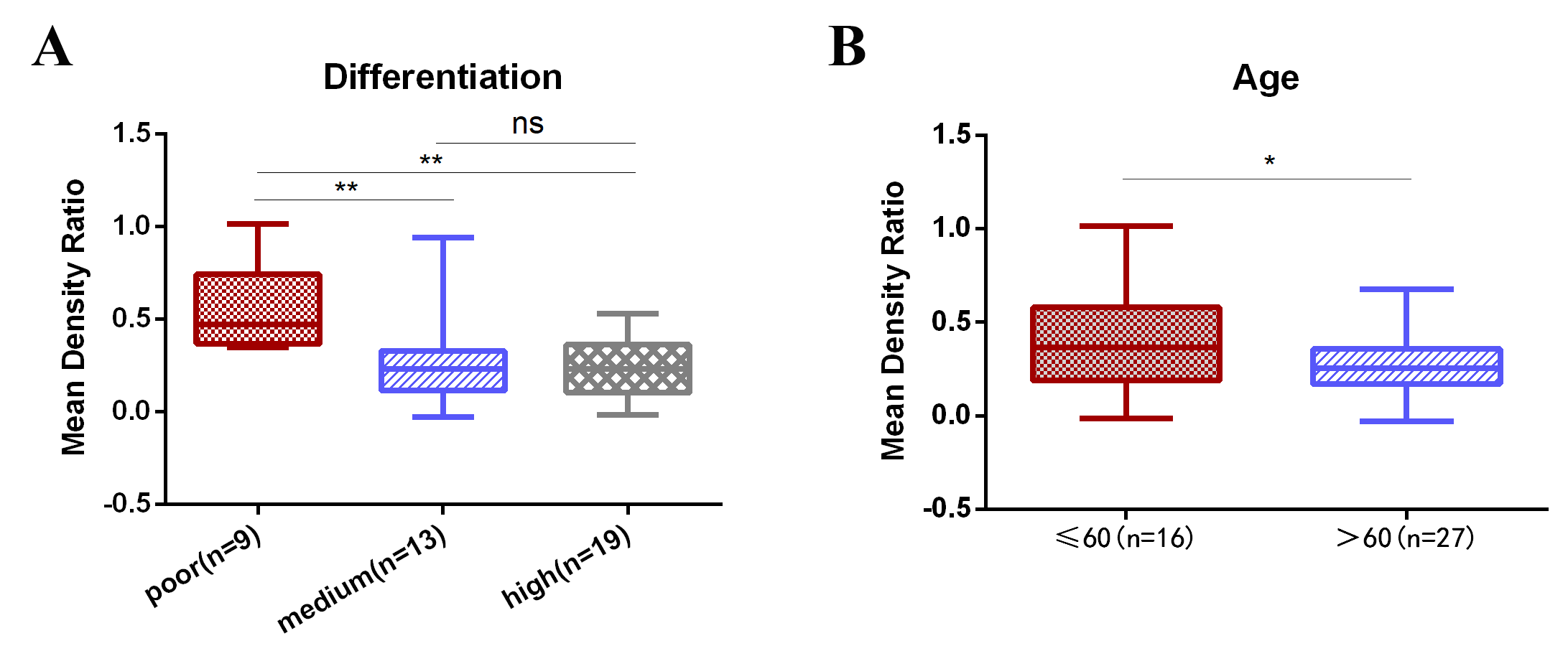

Supplement: Supplementary Figure 1 — Relationship between WIPI2 and clinicopathological features (A) Relationship between the relative expression levels of WIPI2 and the degree of differentiation of colorectal cancer patients. (B) Relationship between the relative expression levels of WIPI2 and the age of colorectal cancer patients. **P < 0.01. [file Image_1.tif]
