# Supplementary material for: WIPI2 enhances the vulnerability of colorectal cancer cells to erastin via bioinformatics analysis and experimental verification
Source: Front Oncol. 2023 May 3;13:1146617. doi: 10.3389/fonc.2023.1146617 (PMC10189881; doi:10.3389/fonc.2023.1146617)
Supplement: Supplementary Table 1 — One-way COX regression analysis of ferroptosis-associated genes. [file Table_1.docx]

| id | HR | HR.95L | HR.95H | pvalue |
| --- | --- | --- | --- | --- |
| ADAMTS13 | 1.60 | 1.06 | 2.42 | 0.03 |
| ADIPOQ | 1.31 | 1.02 | 1.67 | 0.03 |
| ALOX12 | 2.20 | 1.18 | 4.08 | 0.01 |
| ATG13 | 2.28 | 1.19 | 4.38 | 0.01 |
| BRD3 | 1.78 | 1.04 | 3.07 | 0.04 |
| BRDT | 6.56 | 1.29 | 33.29 | 0.02 |
| CARS1 | 1.99 | 1.10 | 3.61 | 0.02 |
| CDH1 | 0.68 | 0.49 | 0.95 | 0.02 |
| CDKN2A | 1.26 | 1.05 | 1.51 | 0.01 |
| CISD2 | 0.48 | 0.27 | 0.84 | 0.01 |
| COX4I2 | 1.76 | 1.23 | 2.52 | 0.00 |
| DUOX1 | 1.55 | 1.01 | 2.37 | 0.04 |
| EGFR | 1.45 | 1.03 | 2.04 | 0.03 |
| ENO3 | 2.15 | 1.35 | 3.42 | 0.00 |
| FABP4 | 1.20 | 1.05 | 1.36 | 0.01 |
| FNDC5 | 2.19 | 1.39 | 3.43 | 0.00 |
| FZD7 | 1.26 | 1.01 | 1.59 | 0.04 |
| GSTM1 | 1.24 | 1.04 | 1.48 | 0.01 |
| HOTAIR | 1.50 | 1.11 | 2.04 | 0.01 |
| IFNA1 | 107.11 | 1.29 | 8879.46 | 0.04 |
| LINC00336 | 4.12 | 1.31 | 12.95 | 0.02 |
| MAPK9 | 0.47 | 0.23 | 0.97 | 0.04 |
| MIR9-3HG | 17.19 | 5.41 | 54.57 | 0.00 |
| MPC1 | 0.60 | 0.41 | 0.86 | 0.01 |
| MYB | 0.75 | 0.57 | 0.97 | 0.03 |
| NOS2 | 0.86 | 0.75 | 0.99 | 0.03 |
| NOX4 | 1.59 | 1.02 | 2.47 | 0.04 |
| PANX2 | 1.58 | 1.06 | 2.35 | 0.03 |
| PARP10 | 1.44 | 1.05 | 1.99 | 0.03 |
| PARP15 | 1.74 | 1.02 | 2.96 | 0.04 |
| PDSS2 | 0.47 | 0.27 | 0.81 | 0.01 |
| PHF21A | 2.17 | 1.18 | 3.98 | 0.01 |
| PPP1R13L | 1.35 | 1.00 | 1.81 | 0.05 |
| PTPN6 | 2.04 | 1.25 | 3.33 | 0.00 |
| SLC39A14 | 0.67 | 0.48 | 0.93 | 0.02 |
| TERT | 2.04 | 1.26 | 3.30 | 0.00 |
| TIMP1 | 1.39 | 1.08 | 1.78 | 0.01 |
| TRIB2 | 1.29 | 1.03 | 1.61 | 0.03 |
| ULK1 | 1.70 | 1.08 | 2.66 | 0.02 |
| WIPI1 | 0.71 | 0.51 | 0.99 | 0.05 |
| WIPI2 | 1.96 | 1.08 | 3.54 | 0.03 |
| WWTR1 | 1.32 | 1.04 | 1.67 | 0.02 |

**Table S1: One-way COX regression analysis of ferroptosis-associated genes**
