# Supplementary material for: WIPI2 enhances the vulnerability of colorectal cancer cells to erastin via bioinformatics analysis and experimental verification
Source: Front Oncol. 2023 May 3;13:1146617. doi: 10.3389/fonc.2023.1146617 (PMC10189881; doi:10.3389/fonc.2023.1146617)
Supplement: Supplementary Table 2 — Correlation between clinical pathological features and the expression of WIPI2 in colorectal cancer patients. [file Table_2.docx]

| **Table S2 Correlation between clinical pathological features and the expression of WIPI2 in colorectal cancer patients** | | | |
| --- | --- | --- | --- |
| **Clinical features** | **Groups** | **Cases** | **p value** |
| Age（years） | ≦60 | 16 | 0.046 |
|  | ﹥60 | 27 |  |
| Gender | Male | 19 | 0.9368 |
|  | Female | 24 |  |
| Degree of differentiation | Poor | 9 | 0.0011 |
|  | Medium | 13 |  |
|  | High | 19 |  |
| Tumor size (cm) | ﹤5 | 24 | 0.483 |
|  | ≧5 | 17 |  |
| Clinical stages | Ⅱ | 25 | 0.6677 |
|  | Ⅲ | 13 |  |
|  | Ⅳ | 3 |  |
| Lymph node metastasis | Positive | 16 | 0.8632 |
|  | Negative | 26 |  |
